# Effects of Sterilization Cycles on PEEK for Medical Device Application

**DOI:** 10.3390/bioengineering5010018

**Published:** 2018-02-21

**Authors:** Amit Kumar, Wai Teng Yap, Soo Leong Foo, Teck Kheng Lee

**Affiliations:** College Central, Institute of Technical Education, 2 Ang Mo Kio Drive, Singapore 567720, Singapore; amittonk@gmail.com (A.K.); yap_wai_teng@ite.edu.sg (W.T.Y.); foo_soo_leong@ite.edu.sg (S.L.F.)

**Keywords:** polyetheretherketone, PEEK, DSC, Vickers hardness, medical grade, medical device

## Abstract

The effects of the sterilization process have been studied on medical grade thermoplastic polyetheretherketone (PEEK). For a reusable medical device, material reliability is an important parameter to decide its lifetime, as it will be subjected to the continuous steam sterilization process. A spring nature, clip component was selected out of a newly designed medical device (patented) to perform this reliability study. This clip component was sterilized for a predetermined number of cycles (2, 4, 6, 8, 10, 20…100) at 121 °C for 30 min. A significant decrease of ~20% in the compression force of the spring was observed after 30 cycles, and a ~6% decrease in the lateral dimension of the clip was observed after 50 cycles. No further significant change in the compression force or dimension was observed for the subsequent sterilization cycles. Vickers hardness and differential scanning calorimetry (DSC) techniques were used to characterize the effects of sterilization. DSC results exhibited no significant change in the degree of cure and melting behavior of PEEK before and after the sterilization. Hardness measurement exhibited an increase of ~49% in hardness after just 20 cycles. When an unsterilized sample was heated for repetitive cycles without the presence of moisture (121 °C, 10 and 20 cycles), only ~7% of the maximum change in hardness was observed.

## 1. Introduction

In the late 1990s, polyetheretherketone (PEEK) was initially developed as a high-performance thermoplastic to replace metal-based orthopedic [1,2] and trauma [3,4] implants. Invibio Ltd. (Thornton Cleveleys, United Kingdom) first offered PEEK commercially as a biomaterial for implant applications in 1998, and since then research on PEEK as a biomaterial has enhanced significantly [5]. For implant applications, many PEEK composite materials have been developed, which have been studied for their behavior under mechanical impact, biotribology, friction, dynamic, damage, and fracture [6,7,8,9,10].

A thermomechanical study performed on PEEK composite [11,12] reported the changes in crystallinity, macroscopic decoloration, large deformation in impact, high strain rate, and heating-induced deformation. As all implants were subjected to body temperature, which is significantly below the glass transition temperature of PEEK, no significant change in elastic properties of PEEK was observed [13]. However, it was reported that the yielding and plastic flow behavior of PEEK was affected at physiological temperatures [12,14].

For a medical device, which will be used in surgery, the steam sterilization process is mandatory to properly disinfect the device before its next use. The influence of the sterilization process on the micromechanical properties of carbon fiber-reinforced PEEK has been studied [15] for bone implant applications. It was reported that after 3 steam sterilization cycles, there was no significant change in elastic modulus, hardness, or coefficient of friction of carbon composite PEEK. The influences of thermal cycling (temperature range between +60 °C and −60 °C, 750 cycles) on the fatigue behavior of carbon/PEEK laminates were also studied [16]. No significant change in tensile properties was observed, but a decrease of 25% in fatigue strength was observed. In another study, the fatigue performance of PEEK was investigated under sterilization and thermal aging cycles and no significant change in fatigue performance of PEEK was found [17]. Most of these studies performed on PEEK are basically for implant applications, low temperature thermal cycling fatigue, or to investigate the behavior after only a few cycles of sterilization. However, PEEK should be investigated for a higher number of sterilization cycles in order to test its reliability in surgical device applications.

In this work, the thermal reliability of medical grade PEEK (natural) was investigated for its use in surgical device applications. Although there are many available medical grade plastic materials, such as PTFE (polytetrafluoroethylene), PEEK, PC (polycarbonate), PS (polysulfone), and PVDF (polyvinylidene fluoride), PEEK presents the best combination of mechanical (stiffness, hardness, and wear) and thermal (high glass transition and melting temperature) properties required for the dimensional and thermal stability of a medical device [18]. An important reason to choose natural PEEK is its radiolucent property [13], which makes it transparent to radiography. If any surgical device is fabricated using natural PEEK, then the real-time monitoring of surgery is possible, as X-ray can clearly see through this material.

## 2. Materials and Methods 

In this work, two different medical grade PEEK materials (SUSTAPEEK MG NAT 1000X ϕ-12 mm and ϕ-50 mm from Roechling, Germany and TECAPEEK MT natural rod, ϕ-50mm from Ensinger, Germany) were used to fabricate samples for thermal reliability studies. Both materials were of medical grade natural PEEK, having nearly similar mechanical and thermal properties, except the water absorption value which was 0.2% for Roechling PEEK and 0.02% for Ensinger PEEK in 24 h at 23 °C [19,20]. 

### 2.1. Specimen Preparation

**Figure 1:**
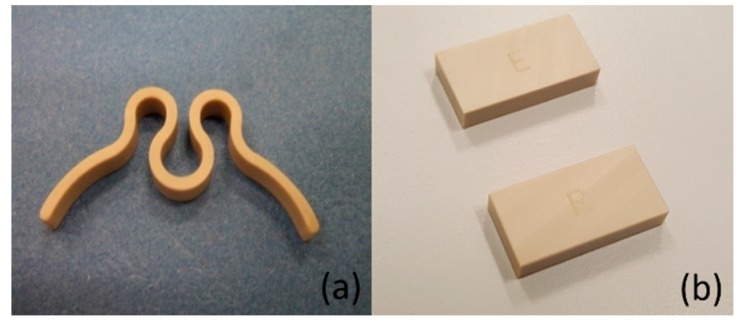
(**a**) Spring-type clip component of a medical device and (**b**) fabricated test specimens for hardness and differential scanning calorimetry (DSC) characterization.

Figure 1a shows a picture of the spring-type clip component part of a medical device [21], which is manufactured from Roechling PEEK using the machining process. Figure 1b shows the image of test samples used for the hardness and differential scanning calorimetry (DSC) characterization, which are also manufactured by the machining process using PEEK materials procured from Roechling and Ensinger. Fifteen samples, each 20 × 10 × 4 mm in size, were fabricated using two different sizes of cylindrical rod, with 12 mm and 50 mm diameters, to investigate the effect of primary processing conditions on the thermal cyclic performance of PEEK during sterilization.

### 2.2. Autoclave Sterilization

A pressure cooker tester (PC-242HS, HIRAYAMA, Tokyo, Japan) autoclave was used to perform the sterilization test on the clip component and test samples. Samples were heated up to 121 °C for 30 min under 0.1 MPa pressure at a nearly 100% humidity level. Initially, the clip component was subjected to 2, 4, 6, 8, 10, 20…90, 100 sterilization cycles, and after each set of cycles (for example after 2 cycles, then after 4 cycles) the clip was tested for the compression force and dimensional change until the 100 cycles were finished. Later, 14 test samples of each type (12 and 50 mm Roechling, 50 mm Ensinger) were placed inside the autoclave and after 2, 4, 6, 8, 10, 20…90, 100 sterilization cycles, one sample of each type was brought out from the autoclave and characterized for hardness and DSC. 

### 2.3. Characterization

#### 2.3.1. Clip Characterization

The clip component, after each set of autoclave cycles (2, 4, 6, 8, 10…90, 100) and before the compression force measurement, was subjected to 250 compression cycles (force, 1 kgf ± 20% or 0.8 to 1.2 kgf) using an in-house designed jig to test the complete device assembly [21]. The detailed calculation to determine the number of clip compression cycles after each set of sterilization cycles and based on its actual usage in the device assembly is included in the supplementary information. To compare the effect of the sterilization cycles on the compression force of the clip, another similar clip was tested directly for compression force after each 250 spring compression cycles without sterilization (up to 6600 cycles). The compression force of the clip, in both the condition with and without sterilization, was measured using a force gauge (DigiTech, Osaka, Japan, Model: DTG-10) with a 100 N force capacity. At the same time, the lateral dimension of the clip was also measured using a digital Vernier caliper. This whole process was repeated until the 100 sterilization cycles were completed. 

#### 2.3.2. Test Samples Characterization

The hardness of the test samples was measured using a Vickers hardness tester VMT-7, an automatic digital hardness tester (MATSUZAWA, Akita Japan) with a square-based pyramid shaped indenter with a phase angle of 136°. An indenting load of 5 Kgf was used for a dwell time of 15 s. The testing was performed as per ASTM E384-11e1. Each sample was indented for 10 indentations and the average of these values is considered as the hardness of the sample. 

The DSC test was performed using professional Q20 equipment from TA Instruments, New castle, DE, USA. Small chips from the test samples were sliced using wire cutting pliers. A small quantity (< 20 mg) of chipped PEEK material was placed inside a hermetic Al pan, which was sealed with an Al pan cover. DSC characterization was performed between the temperature range from 40 °C to 400 °C with a ramp rate of 20 °C/min, as per ISO 11357 standard. A continuous flow of argon gas at 50 mL/min was maintained throughout the DSC testing. 

## 3. Results

### 3.1. Compression Force and Dimension

**Figure 2:**
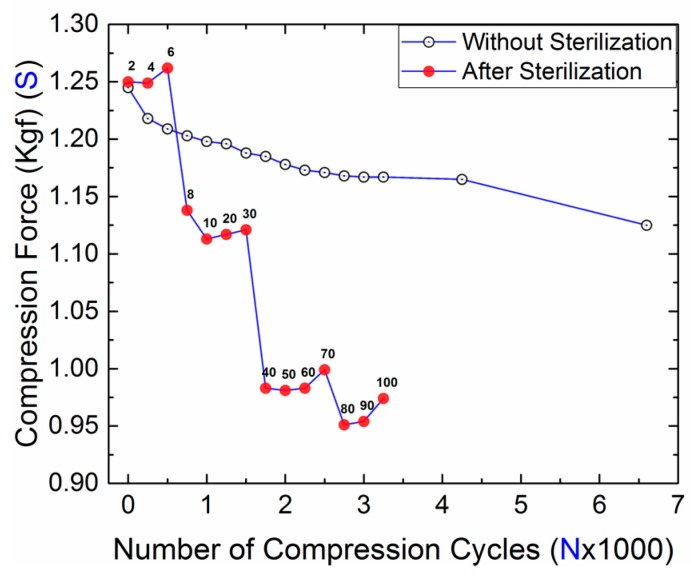
Plot between the compression force and the number of compression cycles of the clip with and without sterilization cycles.

Table 1 consists the results of the compression force and clip dimensions after each set of autoclave cycles (2, 4, 6…90, 100). It was observed that the sterilization has no significant effect on the compression force of the clip up to 4 cycles; this is in good agreement with the work of Godara et al. [15] on sterilization effects on carbon composite PEEK. From 6 to 20 cycles there was a decrease of ~10% in the compression force, which further decreased up to ~20% for 30 cycles. After 30 cycles, the change in compression force became insignificant. A similar trend was observed in the clip dimension results; first, the clip size decreased up to 40 cycles and then the size became stable. Figure 2 shows the plot between the compression force and the number of clip compression cycles with and without sterilization cycles. From the results, it can be seen that the sterilized sample exhibited a sudden decrease in the compression force of the clip after every few sterilization cycles, whereas the results for the unsterilized sample showed a gradual decrease in the compression force of the clip. This difference in behavior of the compression force for both clip samples is due to the absorption of moisture during the sterilization process, which induces stresses due to the expansion and results in the degradation of the structural properties [22]. In general, moisture absorption in polymer occurs through diffusion and capillary processes [23,24,25], which induce plastic deformation either by plasticization or by differential strain due to the swelling while stretching the polymeric chains [26]. These effects can significantly alter the physical, chemical, or mechanical characteristics of materials at different scales [27]. In the current situation, the clip was subjected to moisture at a high temperature (121 °C). As the PEEK (Roechling) used to manufacture the clip has a moisture absorption capacity of 0.2% in 24 h at 23 °C [20], at the high temperature of sterilization a significant amount of moisture absorption can be expected due to the higher diffusion rate of the moisture. Thus, the change in the compression force behavior of the clip is mainly due to the effects of heat and moisture. 

**Figure 3:**
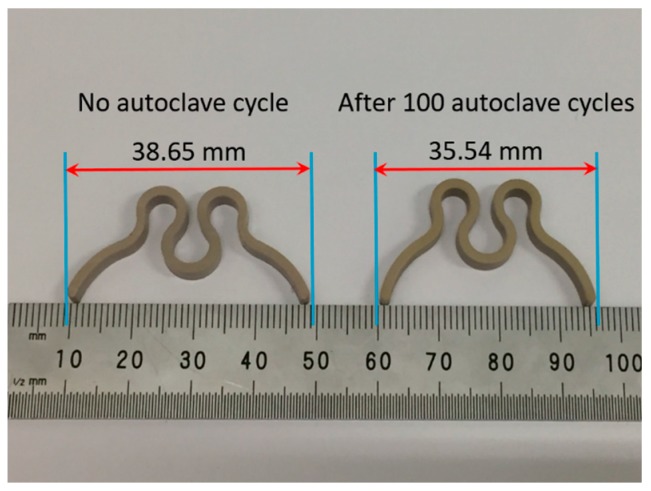
Clip component lateral dimension before and after 100 sterilization cycles.

Figure 3 represents the change in the clip lateral dimension before and after 100 cycles of sterilization. The size of the clip initially decreased faster up to 40 cycles and then slowed down for subsequent cycles, with a maximum of 8% change after 100 sterilization cycles. The spring size is directly related to the spring nature of the clip, as this will bring the clip back to its original size after compression. Therefore, both decreased at the same time due to permanent plastic deformation in the clip material. 

To understand the permanent plastic deformation of the clip material due to the effects of moisture and heat, the rectangular shape (Figure 1b) test samples were characterized for hardness and DSC analysis after a similar set of sterilization cycles. 

### 3.2. Hardness

**Figure 4:**
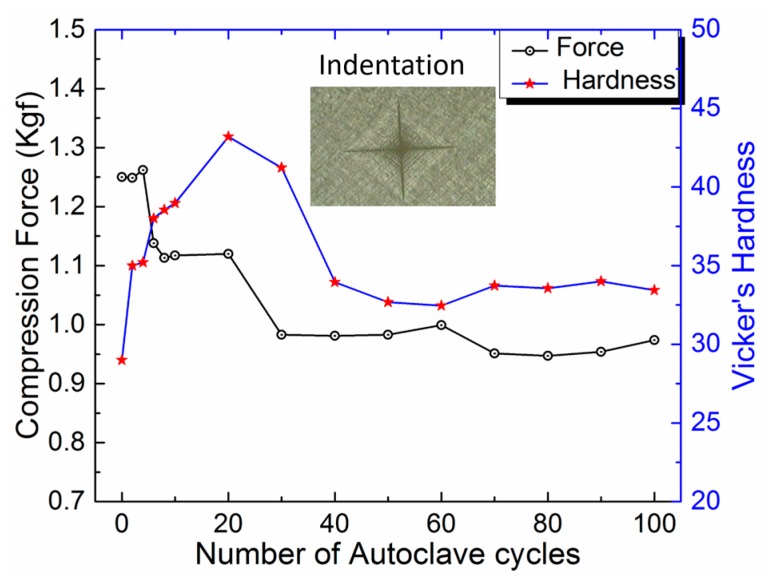
Change in the compression force with the hardness variation of the clip versus the number of autoclave cycles.

Table 1 comprises the hardness characterization results for the test samples made from Roechling PEEK with a 12 mm diameter rod. Figure 4 shows the graphical representation of the change in compression force due to hardness variation of the clip with respect to the number of autoclave cycles. The inset image in Figure 4 shows the indentation cavity formed during the hardness measurement. The hardness results show that the hardness value increased up to ~48% after 20 sterilization cycles and there was a decrease until ~17% after 40 cycles. After 40 cycles, the change in hardness value became stable between 10.34% to 17.24%. These results confirm that there is a change in the material’s mechanical property in terms of hardness scale due to the effects of heat and moisture during the sterilization process. Once the moisture absorption capacity of PEEK reached to its saturation value (at nearly 40 cycles), there were no further changes in the hardness value of the test samples (or in the compression force of the clip). 

**Figure 5:**
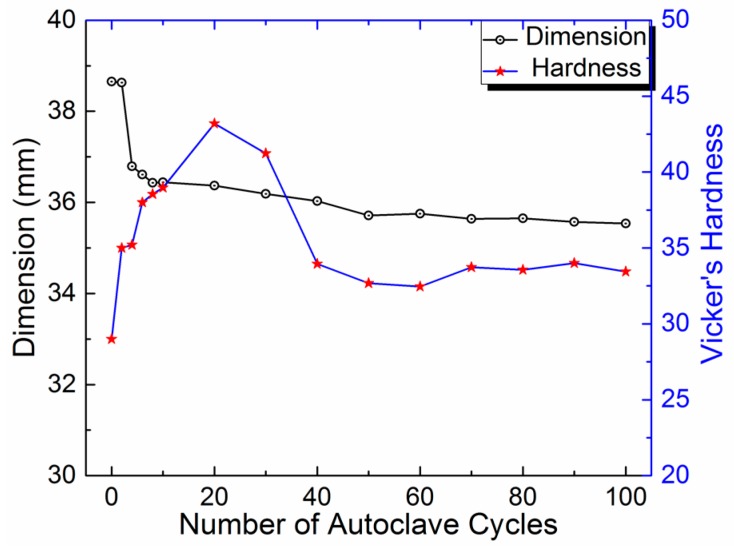
Change in the clip dimension with the hardness variation of the clip with respect to the number of autoclave cycles.

Figure 5 shows the plot between the changes in clip dimension with the hardness variation of the clip with respect to the number of autoclave cycles. A similar type of conclusion can be drawn from these results. The clip size decreased faster for the initial 40 cycles of sterilization and became stable for the subsequent number of autoclave cycles. An increase in the hardness represents a decrease in the elastic nature of the material, and therefore a decrease in the compression force and dimension of the spring due to permanent plastic deformation. 

Table 2 contains the hardness comparison results of the samples made from 12 mm and 50 mm diameter PEEK rods procured from Roechling and Ensinger. It was observed that the sample fabricated from the 12 mm rod was more prone to hardening compared to the sample fabricated from the 50 mm rod. Results show that the sample made from the Roechling 50 mm rod exhibited an increase of ~32% and the sample made from the Ensinger 50 mm rod exhibited an increase of ~25% in hardness compared to the ~48% increase in hardness of the sample made from the Roechling 12 mm rod. This indicates that the initial condition of the raw material, which is being used for the fabrication of device components, is also very important, as it may exhibit different types of material degradation behavior under cyclic sterilization conditions. These changes in the degradation behavior could be due to the different cooling rate for 12 mm and 50 mm rods while they are extruded. The 12 mm rod would have cooled faster as the surface area to volume ratio is higher compared to the 50 mm rod. Due to the faster cooling rate, the 12 mm rod might induce more points and line defects in the material, which cause a deeper and faster diffusion of moisture in the PEEK material, resulting in a faster rate of hardening. 

**Figure 6:**
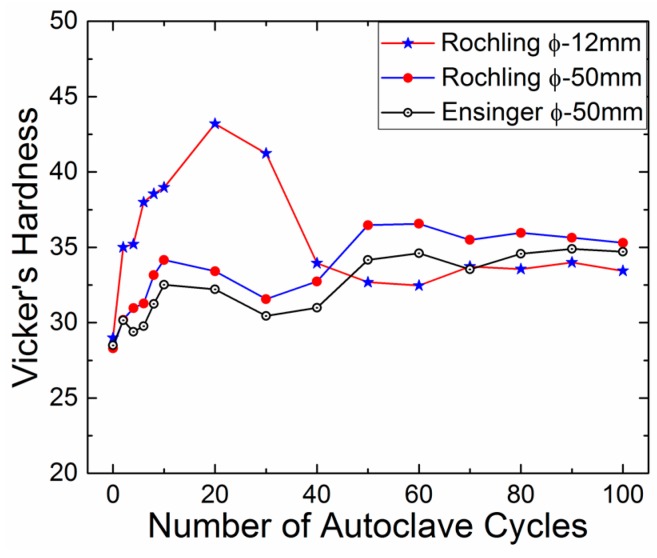
Effect of rod size on the hardness variation with respect to autoclave cycles.

Figure 6 shows the graphic of the hardness variation of different test samples with respect to the number of autoclave cycles. For the first 50 cycles, the rate of hardening was different in the samples fabricated from 12 mm and 50 mm rods. For more than 50 cycles, the variation in hardness value became insignificant, irrespective of the different processing conditions of the raw material (12 mm or 50 mm rods). The slight difference in the hardness value of the samples made from 50 mm Ensinger and Roechling rods was due to their different moisture absorption capacities (0.02% and 0.2% in 24 h at 23 °C) [19,20]. 

### 3.3. Differential Scanning Calorimetry

**Figure 7:**
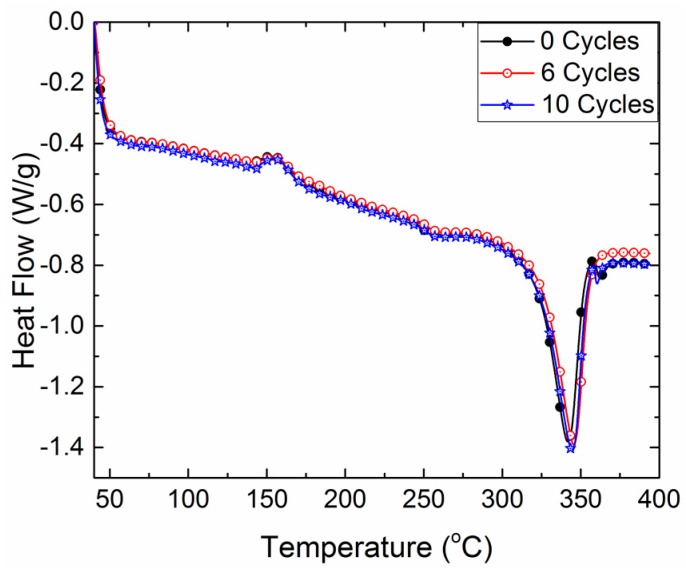
DSC curves between the temperature and heat flow of Roechling PEEK (12 mm) after 0, 6, and 10 autoclave cycles.

Figure 7 shows the DSC testing results after 0, 6, and 10 autoclave cycles for the test sample fabricated from 12 mm Roechling PEEK. Results show that there was no significant change observed in the DSC behavior of PEEK even after 10 sterilization cycles. This observation indicates that the changes in PEEK properties are not due to a change in its bulk material properties, but rather a surface-driven phenomenon due to the diffusion of moisture at high temperatures. Therefore, the thickness of a component plays an important role in deciding the material’s properties and degradation rate to thus decide the component’s life. 

In order to further verify that the degradation of PEEK material properties were due to the diffusion of moisture at a high temperature (121 °C), an untreated test sample (50 mm, Roechling) was repetitively heated (at 121 °C temperature for 30 min) for 10 and 20 cycles and cooled to room temperature, in an electric oven without the presence of moisture. Table 3 lists the results of the hardness measurement after repetitive heating and cooling cycles without the presence of moisture and the hardness of samples that had already undergone a similar number of sterilized cycles. The results show that repetitive heating without moisture exhibited only a 7.14% increase in hardness, irrespective of the number of cycles. Conversely, the hardness change due to repetitive heating in the presence of moisture (sterilization) was measured as 21.43% and 17.86% for the same number of cycles. Another experiment was performed on the sterilized samples, which had already undergone 20 and 30 autoclave cycles. Both samples were heated for a long time (20 h, 100 °C) to remove the moisture content and then the hardness of both the samples was measured again. It was observed that the hardness of the samples after a long time heating was reduced tremendously to a level of 6.89% and 3.45% for 20 and 30 cycles, respectively, compared to 48.28% and 42.38% just after the sterilization cycles (see Table 3). Both of these experiments performed on the test sample revealed the role of moisture content in the hardness increment of PEEK after the sterilization cycles. Therefore, the moisture at higher temperatures during the sterilization process caused a very detrimental effect on the mechanical properties of PEEK polymer due to the high diffusion rate of moisture at high temperatures.

## 4. Conclusions

The effects of the sterilization process on the material properties (reliability) of medical grade thermoplastic polyetheretherketone (PEEK) have been studied. Test results on the clip component of the medical device concluded that there is a decrease of ~20% in the compression force of the clip after 30 autoclave cycles and a decrease of ~6% in the lateral dimension of the clip after 50 autoclave cycles. The change in both the compression force and dimension become stable for the subsequent cycles of sterilization. DSC results on the test sample concluded that there are no significant changes in bulk material properties and the degradation behavior of PEEK properties is a surface-driven phenomenon. Hardness testing results further confirmed that the changes in the clip properties for the initial sterilization (30–50) cycles are due to changes in the hardness or due to the absorption of moisture on the surface of the polymer. When an untreated sample of PEEK was subjected to repetitive heating and cooling cycles in the absence of moisture, it was observed that the hardness of the PEEK material increased by only 7.14% after 10 and 20 cycles, respectively, compared to 21.43% and 17.86% in the presence of both heat and moisture during the sterilization process. When the sterilized samples were heated for a long time (20 h) to remove the moisture content absorbed during sterilization, a tremendous decrease in the hardness value of the samples were observed compare to just after the sterilization hardness. Therefore, it can be concluded that the degradation of PEEK is mainly due to the effect of moisture at elevated temperatures. Thus, this thermal reliability study on PEEK suggests that if a reusable medical device is fabricated using PEEK, which will be subjected to repeated sterilization processes, the change in mechanical properties of PEEK needs to be accounted for in the proposed design.

## Figures and Tables

**Table 1:** Compression force and dimension change results of the clip component in comparison with number of autoclave cycles and test sample’s hardness measurement.

Autoclave Cycles	Compression Force (Kgf)	Dimension Change (mm)	Hardness Roechling (ϕ-12 mm)
	Value (% Change)	Value (% Change)	Value (% Change)
0	1.250	38.65	29 ± 0.8
2	1.249 (↓0.08)	38.63 (↓0.05)	35 ± 1.6 (↑20.69)
4	1.262 (↓0.96)	36.79 (↓4.81)	35 ± 1.4 (↑20.69)
6	1.138 (↓8.96)	36.61 (↓5.28)	38 ± 2.0 (↑31.03)
8	1.113 (↓10.96)	36.43 (↓5.74)	39 ± 1.8 (↑34.48)
10	1.117 (↓10.64)	36.44 (↓5.72)	39 ± 3.7 (↑34.48)
20	1.120 (↓10.4)	36.37 (↓5.90)	43 ± 1.6 (↑48.28)
30	0.983 (↓21.36)	36.19 (↓6.36)	41 ± 2.3 (↑41.38)
40	0.981 (↓21.52)	36.03 (↓6.78)	34 ± 1.7 (↑17.24)
50	0.983 (↓21.36)	35.71 (↓7.61)	33 ± 1.6 (↑13.79)
60	0.999 (↓20.08)	35.75 (↓7.50)	32 ± 2.0 (↑10.34)
70	0.951 (↓23.92)	35.64 (↓7.79)	34 ± 2.0 (↑17.24)
80	0.947 (↓24.24)	35.65 (↓7.76)	34 ± 1.2 (↑17.24)
90	0.954 (↓23.68)	35.57 (↓7.97)	34 ± 1.4 (↑17.24)
100	0.974 (↓22.08)	35.54 (↓8.05)	33 ± 1.3 (↑13.79)

**Table 2:** Comparison of the hardness values of test samples manufactured from 12 mm and 50 mm rods and from two different PEEK manufacturers.

Autoclave Cycles	Hardness Roechling (ϕ-12 mm)	Hardness Roechling (ϕ-50 mm)	Hardness Ensinger (ϕ-50 mm)
	Value (% Change)	Value (% Change)	Value (% Change)
**0**	29 ± 0.8	28 ± 1.2	28 ± 0.6
**2**	35 ± 1.6 (↑20.69)	30 ± 1.2 (↑7.14)	30 ± 1.0 (↑7.14)
**4**	35 ± 1.4 (↑20.69)	31 ± 1.0 (↑10.71)	29 ± 1.1 (↑3.57)
**6**	38 ± 2.0 (↑31.03)	31 ± 1.0 (↑10.71)	30 ± 1.7 (↑7.14)
**8**	39 ± 1.8 (↑34.48)	33 ± 0.7 (↑17.86)	31 ± 1.7 (↑10.71)
**10**	39 ± 3.7 (↑34.48)	34 ± 0.9 (↑21.43)	33 ± 2.2 (↑17.86)
**20**	43 ± 1.6 (↑48.28)	33 ± 2.2 (↑17.86)	32 ± 1.6 (↑14.29)
**30**	41 ± 2.3 (↑41.38)	32 ± 1.4 (↑14.29)	30 ± 1.5 (↑7.14)
**40**	34 ± 1.7 (↑17.24)	33 ± 1.2 (↑17.86)	31 ± 1.8 (↑10.71)
**50**	33 ± 1.6 (↑13.79)	37 ± 1.4 (↑32.14)	34 ± 1.9 (↑21.43)
**60**	32 ± 2.0 (↑10.34)	37 ± 2.5 (↑32.14)	35 ± 2.0 (↑25)
**70**	34 ± 2.0 (↑17.24)	35 ± 1.0 (↑25)	34 ± 1.2 (↑21.43)
**80**	34 ± 1.2 (↑17.24)	36 ± 1.5 (↑28.57)	35 ± 1.8 (↑25)
**90**	34 ± 1.4 (↑17.24)	36 ± 0.9 (↑28.57)	35 ± 1.3 (↑25)
**100**	33 ± 1.3 (↑13.79)	35 ± 1.4 (↑25)	35 ± 1.7 (↑25)

**Table 3:** Effect of heating without moisture on the hardness of Roechling PEEK test samples.

**Repetitive Heating Cycles without Moisture**			
No. of cycles	Hardness Roechling (50 mm) MPa	Hardness Sterilized sample MPa (%)	Hardness heated sample MPa (%)
10	28 ± 1.2	34 ± 0.9 (21.43↑)	30 ± 1.3 (7.14↑)
20		33 ± 2.2 (17.86↑)	30 ± 1.0 (7.14↑)
**Long Heating Period (20 h, 100 °C) of Sterilized Sample**			
No. of cycles	Hardness Roechling (12 mm) MPa	Hardness Sterilized sample MPa (%)	Hardness heated sample MPa (%)
20	29 ± 0.8	43 ± 1.6 (48.28↑)	31 ± 1.1 (6.89↑)
30		41 ± 2.3 (42.38↑)	30 ± 0.8 (3.45↑)

## Supplementary Materials

The following are available online at http://www.mdpi.com/2306-5354/5/1/18/s1.

Supplementary File 1

## References

[1] Liao K. (1994). Performance characterization and modeling of a composite hip prosthesis. Exp. Tech..

[2] Maharaj G.R., Jamison R.D., Jamison R.D., Gilbertson L.N. (1993). Intraoperative impact: Characterization and laboratory simulation on composite hip prostheses. Composite Materials for Implant Applications in the Human Body: Characterization and Testing.

[3] Kelsey D.J., Springer G.S., Goodman S.B. (1997). Composite implant for bone replacement. J. Compos. Mater..

[4] Corvelli A.A., Biermann P.J., Roberts C. (1997). Design, analysis, and fabrication of a composite segmental bone replacement implant. J. Adv. Mater..

[5] Williams D. (2001). New horizons for thermoplastic polymers. Med. Device Technol..

[6] Wang A., Lin R., Stark C., Dumbleton J.H. (1999). Suitability and limitations of carbon fiber reinforced PEEK composites as bearing surfaces for total joint replacements. Wear.

[7] Jones E., Wang A., Streicher R. Validating the limits for a PEEK composite as an acetabular wear surface. Proceedings of the 27th annual meeting of Society of Biomaterials.

[8] Joyce T.J., Rieker C., Unsworth A. (2006). Comparative in vitro wear testing of PEEK and UHMWPE capped metacarpophalangeal prostheses. Biomed. Mater. Eng..

[9] Manley M., Ong K., Kurtz S.M., Rushton N., Field R.E. Biomechanics of a PEEK horseshoe-shaped cup: Comparisons with a predicate deformable cup. Proceedings of the Transactions of the 53rd Orthopedic Research Society.

[10] Arias A., Rodríguez-Martínez J.A., Rusinek A. (2007). Numerical simulations of impact behaviour of thin steel plates subjected to cylindrical, conical and hemispherical non-deformable projectiles. Eng. Fract. Mech..

[11] Sobieraj M., Rimnac C., Kurtz S. (2012). Fracture, fatigue and noch behavior of PEEK. PEEK Biomaterials Handbook.

[12] El-Qoubaa Z., Othman R. (2015). Characterization and modeling of the strain rate sensitivity of polyetheretherketone’s compressive yield stress. Mater. Des..

[13] Kurtz S., Devine J. (2007). PEEK biomaterials in trauma, orthopedic, and spinal implants. Biomaterials.

[14] Rae P., Brown E., Orler E. (2007). The mechanical properties of poly(ether-ether-ketone) (PEEK) with emphasis on the large compressive strain response. Polymer.

[15] Godara A., Raabe D., Green S. (2007). The influence of sterilization processes on the micromechanical properties of carbon fiber-reinforced PEEK composites for bone implant applications. Acta Biomater..

[16] Tai N.H., Yip M.C., Tseng C.M. (1999). Influences of thermal cycling and low-energy impact on the fatigue behavior of carbon/PEEK laminates. Compos. Part B Eng..

[17] Xin H., Shephered D.E.T., Dearn K.D. (2013). Strength of poly-ether-ether-ketone: Effects of sterilisation and thermal ageing. Polym. Test..

[18] Ensinger Engineering plastics: Manual. https://www.ensingerplastics.com/downloads.

[19] Ensinger TECAPEEK. https://www.ensingerplastics.com/en/shapes/biocompatible-medical-grade/peek.

[20] Roechling SUSTAPEEK. https://www.roechling.com/sg/industrial/materials/thermoplastics/detail/sustapeek-mg-natural-203/.

[21] Leong F.S., Wei N.K., Quan G.J., Hua T.P., Kheng L.T., Hui W.Q., Edmund C., Kesavan E. (2016). System and apparatus for guiding an instrument. U.S. Patent.

[22] Ray B.C. (2006). Temperature effect during humid ageing on interfaces of glass and carbon fibers reinforced epoxy composites. J. Colloid Interface Sci..

[23] Kaelble D.H., Dynes P.J., Maus L. (1976). Hydrothermal aging of compositematerials. 1. Aspects. J. Adhes..

[24] Marom G., Broutman L.J. (1981). Moisture in epoxy resin composites. J. Adhes..

[25] Mijovic M., Lin K.F. (1985). The effects of hygrothermal fatigue on physical mechanical properties and morphology on neat epoxy-resin and graphite composite. J. Appl. Poly. Sci..

[26] Barraza H.J., Aktas L., Hamidi Y.K., Long J., O’Rear E.A., Altan M.C. (2003). Moisture absorption and wet-adhesion properties of resin transfer molded (RTM) composites containing elastomer-coated glass fibers. J. Adhes. Sci. Technol..

[27] Zheng Q., Morgan R.J. (1993). Synergistic thermal—Moisture damage mechanisms of epoxy and their carbon-fiber composites. J. Compos. Mater..

