# Supplementary material for: Effects of Sterilization Cycles on PEEK for Medical Device Application
Source: Bioengineering (Basel). 2018 Feb 21;5(1):18. doi: 10.3390/bioengineering5010018 (PMC5874884; doi:10.3390/bioengineering5010018)
Supplement: Supplementary File 1 [file bioengineering-05-00018-s001.pdf]

bioengineering
MDPI

Supplementary Information

Effects of Sterilization Cycles on PEEK for Medical Device Application

Amit Kumar [1], Wai Teng Yap [1], Soo Leong Foo [1], and Teck Kheng Lee [1,*]

[1] College Central, Institute of Technical education, 2 Ang Mo Kio Drive, Singapore 567720; amittonk@gmail.com
* Correspondence: lee_teck_kheng@ite.edu.sg; Tel.: +65-6580 5007

1. **Calculation for number of compression cycles for 1 year (52 weeks) of usage:**
   Usage of clip component per week: 2 times
   Total usage over 1 year: 52 × 2 = 104 times
   Actual pressing of spring per usage: 2 times
   Possible pressing of spring outside actual usage: 10 times
   Total possible pressing of spring: 104 × 12 = 1248
   Safety factor: 5
   Design cycles: 1248 × 5 = 6240 times
   Declared cycles: 6600 times
2. **Compression cycles after each set of sterilization cycles:**
   After each sterilization cycle, component usage: 5 times
   Safety factor: 5
   Total uses in 10 sterilization cycles: 10 × 5 × 5 = 250 times
